# Natural Product Nitric Oxide Chemistry: New Activity of Old Medicines

**DOI:** 10.1155/2012/873210

**Published:** 2012-04-02

**Authors:** Hong Jiang, Ashley C. Torregrossa, Deepa K. Parthasarathy, Nathan S. Bryan

**Affiliations:** ^1^Texas Therapeutics Institute, Brown Foundation Institute of Molecular Medicine, The University of Texas Health Science Center at Houston, 1825 Pressler Street, Houston, TX 77030, USA; ^2^Department of Integrative Biology and Pharmacology, The University of Texas Graduate School of Biomedical Sciences at Houston, The University of Texas Health Science Center at Houston, 1825 Pressler Street, Houston, TX 77030, USA

## Abstract

The use of complementary and alternative medicine (CAM) as a therapy and preventative care measure for cardiovascular diseases (CVD) may prove to be beneficial when used in conjunction with or in place of conventional medicine. However, the lack of understanding of a mechanism of action of many CAMs limits their use and acceptance in western medicine. We have recently recognized and characterized specific nitric oxide (NO) activity of select alternative and herbal medicines that may account for many of their reported health benefits. The ability of certain CAM to restore NO homeostasis both through enhancing endothelial production of NO and by providing a system for reducing nitrate and nitrite to NO as a compensatory pathway for repleting NO bioavailability may prove to be a safe and cost-effective strategy for combating CVD. We will review the current state of science behind NO activity of herbal medicines and their effects on CVD.

## 1. Introduction

 Complementary and alternative medicine (CAM) is the term for medical products and practices that are not a part of standard care. There is generally a lack of understanding of their mechanisms of action and/or the active compounds. Rigorous, well-designed clinical trials for many CAM therapies are often lacking; therefore, the safety and effectiveness of many of these types of therapies are uncertain and as a result are not recognized as mainstream therapy. However, Traditional Chinese Medicine (TCM) is a form of CAM that remains the primary form of medicine throughout a large portion of Asia and Asian communities in the rest of world with a long history of safety and efficacy in a number of different diseases. In fact, one could argue that TCM is the earliest form of CAM. Defining CAM here in the USA is difficult because the field is very broad and constantly changing. The National Institutes of Health now has a dedicated Center for Complementary and Alternative Medicine (NCCAM) due to the growing popularity of such approaches to ensure safety and promote rigorous clinical trials to demonstrate efficacy. NCCAM defines CAM as a group of diverse medical and health care systems, practices, and products that are not generally considered part of conventional medicine, as practiced by medical doctors and their allied health professionals. The boundaries between CAM and conventional medicine are not absolute, and specific CAM practices may, over time, become widely accepted. Complementary medicine refers to use of alternative treatments together with conventional medicine, such as using acupuncture or herbal medicines in addition to usual care to help with disease management. Most use of CAM by Americans is complementary. Alternative medicine refers to use of CAM in place of conventional medicine. Integrative medicine combines treatments from conventional medicine and CAM for which there is some high-quality evidence of safety and effectiveness. It is also called integrated medicine. Since many CAM-based therapies are derived from TCM, we will provide a brief historical account behind the theory of TCM, and how this has now been integrated into CAM approaches here in the USA.

 TCM has long been used as a major health care system in China and many other countries in Asia. Here in the USA, it is typically referred to as CAM. TCM has its origin in ancient Taoist philosophy, which views a person as an energy system in which the body, mind, and spirit are unified into one when in harmony and are disrupted in disease. TCM practice treats the patient as a whole, not as a part, and it emphasizes a holistic approach that attempts to bring the mind, body, and spirit into harmony. TCM theory is extremely complex and originated thousands of years ago through meticulous observation of nature, the cosmos, and the human body. In TCM theory, imbalance between yin and yang is a summation of all kinds of basic disease and disorders. There is a growing and sustained interest in CAM and TCM fueled by a combination of factors including recognition of the benefits, dissatisfaction with and ineffectiveness of traditional Western medicines, increasing commitment to holistic care, skepticism regarding adverse side effects of drugs, and increasing evidence for the personalized nature of various combinations of herbs for specific disorders [1]. The use of TCM is increasing in Western nations, mostly among people of Southeast Asian origin. Patients who use TCM in Western countries report that the main reason for using it is that TCM is a “more natural” and potentially safer alternative in the treatment of chronic illness than pharmaceutical drugs or surgery [1]. Of the approximately 500 herbs that are in use today, 50 or so are very commonly used alone or in combination. Rather than being prescribed individually, single herbs are combined into formulas that are designed to adapt to the specific needs of individual patients. A herbal formula can contain from 3 to 25 herbs. Each herb has one or more of the four flavors/functions and one of five “temperatures” “*氣*” (pronounced “chi”) (hot, warm, neutral, cool, cold). Herbal formulations work to balance the body from the inside out. Traditional herbal medicines include herbs, herbal materials, herbal preparations, and processed herbal products that contain parts of plants or other plant materials as active ingredients, which assist with strengthening the vital energies (chi), blood, and fluids internally. They are typically administered as tablets, pills, elixirs, soups, liquid extracts, and teas and broadly classified as dietary supplements here in the USA. There are a number of published reports on the association of TCM and NO-related effects [2, 3]. Recently, there are increased interests in the role for NO system in regulation of cardiovascular function by TCM used commonly for cardiovascular disorders. Recognizing and understanding NO activity of TCM may help explain centuries of treatment efficacy in a number of diseases and highlight new treatment options for conditions of NO insufficiency.

## 2. Current Global Markets for CAM

 A 2002 survey of US adults 18 years and older conducted by the National Center for Health Statistics (CDC) and the NCCAM indicated that 74.6% had used some form of CAM, 62.1% had done so within the preceding 12 months, 54.9% used CAM in conjunction with conventional medicine, and 14.8% sought care from a licensed or certified practitioner suggesting that most individuals who use CAM prefer to treat themselves [4]. The industry of alternative and complementary medicines broadly classified as dietary supplements is expected to be $250 billion by 2016 worldwide. In 2004, a survey of nearly 1,400 US hospitals found that more than one in four offered alternative and complementary therapies such as acupuncture, homeopathy, and massage therapy. Herbal treatments are the most popular form of traditional medicine or CAM and are highly lucrative in the international marketplace. Annual revenues in Western Europe reached $5 billion in 2003-2004. In China, sales of products totaled $14 billion in 2005. Herbal medicine revenue in Brazil was $160 million in 2007, according to WHO Fact Sheet no. 134, 2008. Therefore, this industry and type of health care can no longer be ignored. In order to better understand the mechanism of action and the safety profile of herbal remedies, more research and resources are needed. Identifying physiological systems or molecular targets that may be affected by TCM will help propel the field and industry forward and create a better safety and efficacy profile of certain CAMs.

## 3. Production and Regulation of NO in the Cardiovascular System

 Appropriate levels of NO production are critical in tissue blood perfusion and protection of cardiovascular tissues against ischemia and infarction. NO is endogenously generated from the amino acid L-arginine and molecular oxygen in reactions catalyzed by a family of enzymes called NO synthases (NOS). There are three mammalian NOS isoforms: neuronal (nNOS), endothelial (eNOS), and inducible (iNOS). They share 50–60% homology at the amino acid level [5]. Under physiological conditions, the dominant NOS isoform in the vasculature is eNOS, which rather than being a constitutive enzyme as was first suggested is dynamically regulated at the transcriptional, posttranscriptional, and posttranslational levels [6]. Endothelial dysfunction arises from downregulation of eNOS expression and activity and uncoupling of NOS generating free radicals [7]. We now recognize and appreciate that the endothelial production of NO declines progressively with age and can be further reduced by poor diet and lifestyle [8–10]. This becomes the basis for endothelial dysfunction and the etiology of a number of CVD-related symptoms. Restoring endogenous production of NO or providing an exogenous source of NO would be an attractive therapeutic option if this intervention could slow down the progression of endothelial dysfunction and reduce the risk of CVD.

**Figure 1:**
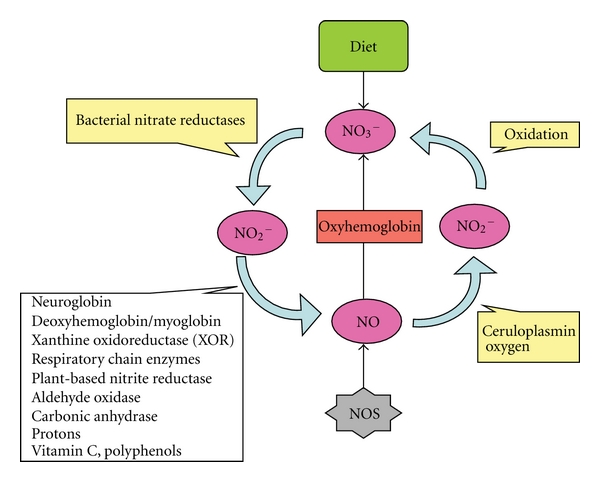
A schematic presentation of a mammalian nitric oxide (NO) cycle. NO is generated by nitric oxide synthases (NOS) in most cells of the body and participates in regulation of numerous physiologic functions. The bioactivity of nitric oxide is partly regulated by its rapid oxidation to nitrite (NO_2_^–^) or, in the presence of oxyhemoglobin, to nitrate (NO_3_^−^). Nitrate is the predominant nitric oxide oxidation product in the circulation. In our bodies, nitrate can undergo reduction to nitrite, and this process is strongly dependent on oral commensal bacteria. In blood and tissues, nitrite can be further reduced to nitric oxide and other bioactive nitrogen oxides. There are several enzymatic and nonenzymatic routes that can catalyze this reduction, most of which are greatly enhanced under hypoxic conditions. This mammalian nitrogen cycle can be fueled by the diet because vegetables contain high amounts of inorganic nitrate.

 Recently, an alternative pathway for NO generation was discovered, wherein the inorganic anions nitrate and nitrite, most often considered inert end products from NO oxidation, can be reduced back to NO and other bioactive nitrogen oxide species. This nitrate-nitrite-nitric oxide pathway is regulated differently than the classic L-arginine-nitric oxide synthase pathway as shown in Figure 1, and it is greatly enhanced during hypoxia and acidosis. Nitrite and nitrate have now moved to the forefront of NO biology [11] with the discovery that it represents a major storage form of NO in both blood and tissues [12]. Dietary nitrite and nitrate have been shown to protect from tissue injury and restore NO homeostasis in eNOS−/− mice [13, 14]. Orally administered nitrite also attenuates cardiac allograft rejection in rats [15]. Nitrate is the primary anion present in green leafy vegetables and has been hypothesized to account for some of the health benefits of vegetables [16]. There are a number of published studies to support this hypothesis via its reduction to nitrite [17, 18]. The nitrate-nitrite-nitric oxide pathway is boosted by dietary intake of nitrate. Dietary nitrate supplementation has been shown to reduce diastolic blood pressure [19], inhibit platelet aggregation, and protect the heart from ischemia-reperfusion injury [20]. This pathway may also explain the effects of certain ethnic diets. Dietary nitrate in Japanese food was shown to lower blood pressure in healthy volunteers [21]. It is well established that diets rich in fruit and vegetables (e.g., the Mediterranean diet) protect against development of cardiovascular disease [22–24] and these data may provide evidence as to why.

 The first metabolic activation step for this pathway requires commensal bacteria to reduce nitrate to nitrite [25]. There are a number of endogenous systems in mammals capable of reducing nitrite to NO although most are very inefficient and inhibited by oxygen [26]. For this system to proceed, there must be enough substrate for reduction and the necessary bacteria and reductive machinery to reduce nitrate all the way down to NO. Dietary and enzymatic sources of nitrate are a potentially large source of nitrite and ultimately NO in the human body. The nitrite converted from nitrate by oral bacteria disproportionates with formation of NO after entering the acidic environment of the stomach, helping to reduce gastrointestinal tract infection, increase mucous barrier thickness and increase gastric blood flow [27]. In addition to the simple protonation of nitrite in the stomach, there are several enzymatic pathways for conversion of systemic nitrite to NO and other bioactive nitrogen species. Hemoglobin, myoglobin, neuroglobin, xanthine oxidoreductase, aldehyde oxidase, carbonic anhydrase, and mitochondrial enzymes have all been identified with having a role in nitrite bioactivation [28] (Figure 1). However, all of these pathways are grossly inefficient along the physiological oxygen gradient with oxygen being a potent inhibitor of nitrite reduction [26, 29]. Therefore, for this system to generate enough bioactive NO, there must be sufficient nitrate and nitrite available for this inefficient reduction. A consequence of endothelial dysfunction is reduced blood and tissue levels of nitrite available for reduction [14, 30], further comprising this alternative NO pathway. Since most of the TCMs are herbal extracts, we initially thought they may be good sources of nitrite and nitrate. Also antioxidants and polyphenols have been shown to effectively reduce nitrite to NO [31]. This combination of nitrite, nitrate and polyphenols could provide then a system for repleting NO homeostasis. We recently tested and confirmed this hypothesis [32] and find that select herbal extracts contain high amounts of nitrate and also the capacity to reduce nitrite to NO. If our current paradigm is true, then restoration of NO homeostasis may be a primary target for treating and preventing CVD. A short review of this paradigm follows.

## 4. NO Insufficiency Is the Root Cause of CVD

 CVD is a group of disorders including congestive heart failure (CHF) and ischemic heart disease that are becoming the leading cause of morbidity and mortality in the world [33, 34]. CVD can result from a quantitative or functional NO deficiency that can limit NO-dependent signal transduction pathways to the detriment of normal cellular function. NO formation by endothelial NO synthase (eNOS) plays an important role in the regulation of vasomotor tone in the pulmonary and systemic vascular beds [35–37]. However, NO generation by eNOS may be rapidly depleted in ischemic conditions, since eNOS is dependent upon the availability of oxygen [38, 39]. Loss of endogenous NO activity has a number of detrimental actions, most notably, vasoconstriction, increased activity and adherence of platelets, and accumulation of inflammatory cells at sites of endothelial damage. Endothelial damage is associated with most forms of CVD. NO possesses a number of physiological properties that make it a potent cardioprotective-signaling molecule [40]. First, NO is a potent vasodilator [41], which allows for regulation of blood flow and essential perfusion of tissue as needed. Secondly, NO reversibly inhibits mitochondrial respiration [42]. The inhibition of mitochondrial respiration during an ischemia-reperfusion event such as heart attack or stroke counterintuitively leads to a decrease in mitochondrial-driven injury by extending the zone of adequate tissue cellular oxygenation away from vessels [43]. Thirdly, NO is a potent inhibitor of neutrophil adherence to vascular endothelium [44]. Neutrophil adherence is an important event initiating further leukocyte activation and superoxide radical generation, which in turn leads to injury to the endothelium and perivascular myocardium in the ischemic heart [45]. Fourth, NO also prevents platelet aggregation [46], which together with the antineutrophil actions of NO attenuates capillary plugging [47]. Finally NO inhibits apoptosis directly or indirectly by inhibiting caspase-3-like activation via a cGMP-dependent mechanisms and/or through protein S-nitrosylation [48, 49]. Without sufficient NO production, the body loses its ability to regulate and control normal vascular function. Therefore, strategies designed to enhance NO production and reduce reactive oxygen species production will likely limit injury and improve recovery from CVD or perhaps even prevent onset and progression of CVD.

## 5. Therapeutic Effects and Nitric Oxide Bioactivity of TCM

 Traditional herbal medicines used for thousands of years in Asia and other regions have been proven effective in certain cardiovascular disorders. Some of the herbal medicines have profound NO bioactivity primarily due to the nitrate-nitrite-NO reduction pathway [32]. They contain very large amounts of nitrate/nitrite in the extracts given to patients [32]. The described benefits of these ancient medications may be attributed to their inherent nitrate/nitrite content combined with their robust nitrate/nitrite reductase activity to generate NO independent of the L-arginine-NO pathway [14, 26, 32, 50]. The first use of nitrate for treatment of patients with symptoms that appears to be angina was described in an 8th century Chinese manuscript uncovered at the Buddhist grotto of Dunhuang [51]. The patients were instructed to take Xiao Shi Xiong Huang San, hold it under the tongue for a time, and then swallow the saliva. The significance of the instructions is that under the tongue, even in a healthy mouth, nitrate-reducing bacteria convert some of the nitrate into nitrite. Therefore, if the patient follows the physician's instructions fully, he or she will be taking nitrite, known to be effective in alleviating pain resulting from angina. Chinese physicians in traditional medicine have more recently tested the therapeutic effects of Xiao Shi Xiong Huang San (the Nitrum and Realgar Powder), one of the Dunhuang prescriptions, on angina pectoris caused by coronary heart disease. Compared to nitroglycerin, Xiao Shi Xiong Huang San showed much higher efficacy and improvement in a clinical trial of 61 patients [52]. Recently, the research results demonstrate that certain TCM increased NO release in rat vascular endothelial cells under hypoxia [53]. Purified ginsenoside Rg1 enhanced the production of NO from IFN-gamma-activated-macrophage cells RAW 264-7 [54]. Mu-Fang-Ji-Tang (TJ-36), a traditional Chinese herbal medicine, is made from four natural traditional Chinese drugs: Panax ginseng radix, Cinnamomum cassia, Sinomenum acutum, and Gypsum fibrosum, which is said to have been used for more than 1800 years to treat heart failure in China. Recent studies have shown that Mu-Fang-Ji-Tang (TJ-36) had protective effects against myocardial injury in a murine model of congestive heart failure induced by viral myocarditis [55]. Our studies have provided convincing evidence that many of these TCM contain significant amounts of nitrate and nitrite and also active nitrite reductase activity in certain herbs known to have protective or therapeutic benefits to patients with CVD (Table 1), such as the Danshen Root (radix salviae miltiorrhizae), Sanchi (radix notoginseng), and Hongshen (radix ginseng) [32]. These herbs, with specific indications for cardiovascular disease can generate NO from nitrite, and relax blood vessels. The therapeutic benefits of these herbal medicines are providing an alternative source of NO to patients that may be unable to make NO from L-arginine owing to endothelial dysfunction. There is an endogenous nitrite reductase activity in animal tissues, such as the liver and aorta, but this inherent biological capacity is low (around 1 pmoL/mg protein). The reductase activity in some of these herbal medicines may exceed that detected in the animal tissues [32]. It is estimated that the increased reductase activity may occur by orders of magnitude, almost 1000 times higher than endogenous production of NO. This would equate to 300 nmoles per day of NO from a single herbal preparation. The average NO production in the human body (70 kg) is 1.68 mmoL NO per day (based on an NO production rate of 1 *μ*moL/kg/h). By supplying the exogenous nitrate/nitrite and reductase activities, herbal medicines offer an alternative therapeutic strategy to combat or treat any condition related to NO insufficiency including heart disease and hypertension. Maintaining NO homeostasis requires the repletion of nitrite and nitrate through which the ability to generate NO can be restored to compensate for the inability of the endothelium to convert L-arginine to NO in coronary heart disease. This concept has recently been tested through the development of a rationally designed dietary supplement with natural products selected for their NO activity based on their nitrite, nitrate content, and an oxygen independent nitrite reductase. In a double-blinded placebo-controlled study in patients over the age of 40 with at least 3 cardiovascular risk factors, this type of technology was found to significantly restore plasma levels of nitrite and nitrate, reduce triglycerides by 27%, and modestly reduce blood pressure and C-reactive protein thereby modifying the cardiovascular risk profile of patients after only 30 days [56].

 Recent clinical studies have provided new evidence on Danshen, a commonly used herbal medicine for CVD in China, which may have a similar efficacy to the known NO donor nitroglycerin [57–59]. The extract of *Salviae Miltiorrhiae*, or Danshen in Chinese, contains large amounts of nitrate [32]. The demonstration of beneficial effects of this herb on ischemic diseases offers an alternative avenue for the management of angina pectoris, myocardial infarction, or stroke [60, 61]. Danshen-related Chinese herbal medicines have been widely used for treatment of coronary heart disease in the East, and a clinical trial is on the way in the United States. Danshen is a routine herbal medication for acute angina pectoris. In addition, it may be effective for dyslipidemia, blood hyperviscosity syndrome, peripheral angiopathy (superficial thrombophlebitis, venous thrombosis, allergic arteriolitis), diabetes mellitus, and cirrhosis and is also used for altitude sickness. Experimental studies have shown that Danshen dilates coronary arteries, increases coronary blood flow, and scavenges free radicals in ischemic diseases, reducing cellular damage from ischemia and improving heart functions, remarkably similar to known effects of NO and nitrite [59]. However, the nitrate/nitrite reductase activity in Danshen is relatively low. Often, Danshen is mixed with other herbal products. One of which is an extract of cinnamon or borneol. Borneol is consumed excessively in China and Southeast Asian countries, particularly in a combined formula for preventing cardiovascular disease. Borneol exerts a concentration-dependent inhibitory effect on venous thrombosis [62]. The antithrombotic activity of borneol contributes to its action in combined formula for preventing cardiovascular diseases. Our recent studies have shown that although the natural form of borneol itself contains very little nitrite and nitrate, it displays a potent nitrite reductase activity [32] that when used in combination with nitrite and nitrate-rich DanShen provides the system for generating NO. Another herbal medicine made from the root of *Radix ginseng *may also have synergistic effects with Danshen. Ginseng contains modest amounts of nitrate but has stronger reductase activity [32]. Therefore, this may explain the mechanism behind the theory of synergy of specific combination of herbs.

## 6. Effects of TCM on L-Arginine/eNOS/NO Signaling Pathway

Some TCM may regulate NO production by exerting regulatory effects on the L-arginine/eNOS/NO signaling pathway. Puerarin is a major active ingredient extracted from the traditional Chinese medicine Ge-gen (Radix Puerariae, RP). It has long been used to treat cardiovascular diseases including coronary artery diseases (CAD), arrhythmia, and hypertension. Recent studies have shown that puerarin increases serum nitrite concentrations in rats with myocardial ischemia through the induction of protein expression and activation of eNOS and through Akt/PKB phosphorylation [63]. Tongxinluo (TXL), a mixture of traditional Chinese medicines, can attenuate the no-reflow and ischemia-reperfusion injury in an infarct animal model [64, 65]. TXL is composed of *Radix ginseng, Buthus martensii, Hirudo, Eupolyphaga seu steleophaga, Scolopendra subspinipes, Periostracum cicadae, Radix paeoniae rubra, Semen ziziphi spinosae, Lignum dalbergiae odoriferae, Lignum santali albi, *and *Borneolum syntheticum*. Owing to its efficacy and minimal adverse effects, TXL has been widely used in China to treat patients with acute coronary syndrome. In a 90-minute ischemia and 3-hour reperfusion model, miniature pigs were randomly assigned to treatment with TXL (gavaged 1 hour prior to ischemia); TXL plus H-89 (protein kinase-A inhibitor intravenously infused before ischemia); or TXL plus N(omega)-nitro-L-arginine (L-NNA; an eNOS inhibitor, intravenously administered prior to ischemia). The results of this study demonstrate that TXL treatment can decrease creatine kinase elevation, improve coronary flow, and reduce infarct size. The effects of TXL may be partially abolished by H-89 or completely reversed by L-NNA. In addition, TXL treatment can elevate the kinase activity and expression, evidenced by expression of Thr198 phosphorated-PKA, Ser1179 phosphorated-eNOS (p-eNOS), and Ser635 p-eNOS in the ischemic myocardium. Addition of H-89 diminishes the TXL activities. Thus, pretreatment with a single low loading dose of TXL 1 hour before ischemia reduces the myocardial no-reflow phenomenon and ischemia-reperfusion injury by upregulating the phosphorylation of eNOS at Ser1179 and Ser635, and this effect is partially mediated by the PKA pathway [64]. Recent studies also have shown that Salvianolic acid B (Sal B) and Tanshinone IIA (Tan IIA) are two of the major components in Danshen, have cardioprotective effects in an in vivo myocardial infarction model of C57 mice, have vasodilator action in an ex vivo microartery system through the endothelial nitric oxide synthase (eNOS)/nitric oxide pathway, and are involved in the regulation of the L-arginine/eNOS/NO pathways in human umbilical vein endothelial cells (HUVECs). Both Sal B and Tan IIA inhibited cardiac hypertrophy and infarction sizes and improved cardiac function at 4 weeks after induction of infarction. Furthermore, an eNOS inhibitor (L-NAME) obliterated the observed effects. Sal B and Tan IIA mediated vasodilatation in mice coronaries ex vivo, the effect of which was decreased with either L-NAME or PI3K inhibitor (LY294002). In addition, Sal B and Tan IIA-induced vasodilatation was observed ex vivo in the microvessels of eNOS−/− mice. Sal B and Tan IIA also stimulated eNOS phosphorylation in a concentration- and time-dependent manner in the HUVEC culture, which was diminished by LY294002. In addition, Sal B and Tan IIA were found to stimulate the phosphorylation of AMPK (Thr(172)) and Akt (Ser(473)), while compound C significantly decreased the phosphorylation of Akt (Ser(473)) mediated by both. Finally, Sal B and Tan IIA were found to induce [(3)H]-L-arginine uptake and increase the CAT-1 and CAT-2B mRNA levels in HUVEC culture [66]. It appears that select TCM herbs alone and in combination can restore NO homeostasis both through an endothelium dependent manner by activating the L-arginine pathway and through an endothelium-independent manner via the nitrate-nitrite-NO pathway that may overcome endothelial dysfunction.

## 7. Effects of CAM on Oxidative Stress and Preservation of NO Activity

 In several chronic diseases, free radicals are by-products of abnormal body metabolism and are important factors for late complications and secondary disease especially those related to NO and endothelial dysfunction [67]. There is increasing evidence that in certain pathologic states the increased production and/or ineffective scavenging of reactive oxygen species (ROS) may play a critical role. Medicinal plants are a source for a wide variety of natural antioxidants [68] and my exhibit their effects via several proposed mechanisms, including inhibition of the activities of cyclooxygenase-2 (COX-2) and nuclear factor-Kappa B (NF-*κ*B), inhibition of angiogenesis, and activation of Nrf2-mediated antioxidant signaling [69], all known to interface with the NO pathway. Dang Gui has been shown to hold anti-inflammatory properties and antioxidant activities, especially when used concurrently with other herbs [70]. Dietz and colleagues have reported that the major lipophilic constituent of Dang Gui, Z-ligustilide, reduces oxidative stress through upregulation of antioxidant enzymes such as NQO1, an Nrf2 pathway gene [71]. Also, *A. sinensis *can protect against oxidant injury through elevated glutathione synthesis whose rate limiting step is regulated by the Nrf2-regulated gene *γ*-GCS [72]. Of the different components in Dan Shen, tanshinone IIA has been identified with the most potent antioxidant activity and cytotoxic properties by inducing apoptosis and differentiation in various human cancer cell lines [73]. Tanshinone IIA also displays antioxidant protection against reactive oxygen species (ROS) induced oxidative stress through stress-activated kinases JNKs and p38 MAPK and by an increase in scavenging of oxygen free radicals [74]. Drinking tea is a tradition that began in ancient China over 5000 years ago. Green tea from the leaves of *Camellia sinensis *is the second most consumed beverage in the world, after water. It is a well-studied herb for its effectiveness in chemoprevention, cancer therapy, and other benefits to overall health. The main components of green tea are polyphenols, known as catechins, which account for 30–42% of the solid weight in green tea leaves. The most abundant polyphenol in green tea is (−)-epigallocatechin-3-gallate (EGCG), which is also the most commonly studied green tea component [75]. EGCG contributes to 10–50% of the total polyphenol contents in green tea and appears to possess the strongest antioxidant activity, about 25–100 fold more potent than vitamins C and E [75]. Reducing oxidative stress and promoting endogenous antioxidant enzymatic defense systems very likely provides an additional benefit of traditional medicines. These antioxidants defenses will enhance endogenous NO production by keeping essential cofactors such as BH4 from becoming oxidized and also preserve and prolong NO activity once it is produced by preventing its scavenging by oxygen radicals.

## 8. Conclusion

**Figure 2:**
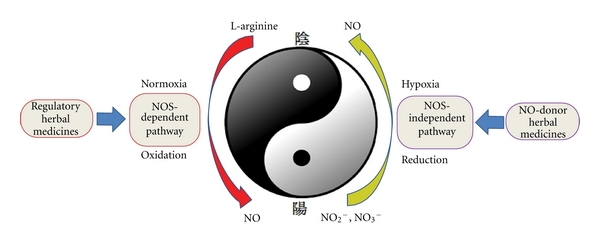
Potential mechanisms of TCM on the regulation of NO Signaling pathway TCM herbs alone and in combination can help restore NO homeostasis in the CVD and maintain “Yin-Yang” balance through reduction of Nitrate/nitrite and oxidation of L-arginine.

 Taken together, the NO signaling pathway plays an important role in the therapeutic effect of many CAMs used for cardiovascular disease (Figure 2). The NO donor-like herbs may serve as an alternative source of nitrate and nitrite or other NO-donors. They contain high activities of nitrite reductase that converts the inorganic anion into NO, which, in turn, relaxes blood vessels and prevents thrombosis. There are also some TCM which contain regulatory factors that influence the expression and activity of eNOS (Figure 2) and may interact with endogenous factors that modulate L-arginine/eNOS/NO signaling pathway. Modern research is providing more evidence to understand specific activity of CAMs that will hopefully provide a mechanistic understanding of their clinical efficacy and allow for better combination of different herbs. However, currently the use of CAM is influenced by legal restrictions, with shifts towards increasing regulation and formal recognition of CAM as means to treat or prevent disease. Further investigation and research are warranted in terms of the functional mechanisms, biosafety, and large-scale clinical trials to firmly establish the efficacy of many of these approaches in the prevention and treatment of illnesses.

## Figures and Tables

**Table 1:** The measurements of nitrite, nitrate, nitroso, nitrite reductase activity in several TCM herbs commonly used for CAD.

Chinese name	English name	Latin name	Indication	Nitrite (ng/g)	Nitrate (mg/g)	Nitroso (nmoL/g)	NO production (pmol/mg)
DanShen	Danahen Root	Radix Salviae Miltiorrhizae	CAD	330	12000	120	7
GuaLou	Snakegourd Fruit	Fructus Trichosanthis	CAD, acute MI, Hyperlipidemia	260	278	120	46
XieBai	Longstamen Onion Bulb	Bulbus Allii Macrostemi	CAD, acute MI, Hyperlipidemia	150	530	842	134
SanChi	Sanchi	Radix Notoginseng	CAD	210	2069	73	13
RuXiang	Frankincense	Resina Olibani	Hypertension	980	61	3210	72
ChiShao	Red Peony Root	Radix Paeonia Rubra	CAD	120	37	450	255
HongSheng	Ginseng	Radix Ginseng	Heart failure, CAD	300	243	76	360
BingPiang	Borneol	Borneolum Syntheticum	Increase other herb's function for CAD or brain disease	120	2.99	6	45
TianRanBingPiang	Borneol	Cinnamomum	Increase other herb's function for CAD or brain disease	160	2.39	0	875
